# Plasma macrophage colony-stimulating factor levels during cardiopulmonary bypass with extracorporeal circulation

**DOI:** 10.1155/S0962935196000518

**Published:** 1996-10

**Authors:** Y. Denizot, P. Fixe, E. Cornu, N. Nathan

**Affiliations:** 1 Department of Anaesthesia CHRU Dupuytren 2 Avenue Martin Luther King Limoges 87042 France; 2 Department of Cardiovascular Surgery CHRU Dupuytren 2 Avenue Martin Luther King Limoges 87042 France

## Abstract

Leukocytosis and thrombocytopenia occur during cardiopulmonary bypass (CPB) with extracorporeal circulation (ECC). Elevated circulating concentrations of macrophage colony-stimulating factor (M-CSF) are reported during thrombocytopenia and leukopenia of different origins. We have assessed M-CSF concentrations in 40 patients undergoing CPB with ECC. Plasma M-CSF concentrations were stable during ECC and increased at the 6th (7.3 ± 0.7 IU/μg protein) and 24th (8.6 ± 0.8 IU/μg protein) postoperative hour compared with pre-ECC values (4.9 ± 0.5 IU/μg protein). A deep thrombocytopenia was found during ECC and until the 24th postoperative hour. A drop of leukocyte counts was found during ECC followed by an increase after ECC weaning. While no correlation was found between M-CSF concentrations and the leukocyte counts, M-CSF values were positively correlated with platelet counts only before and during ECC. Thus, M-CSF is not implicated in the thrombocytopenia and the leukopenia generated during CPB with ECC. However the elevated levels of M-CSFa few hours after the end of ECC might play a role in the inflammatory process often observed after CPB.


Mediators of Inflammation, 5, 358–361 (1996)

### Plasma macrophage colony-stimulating factor levels during cardiopulmonary bypass with extracorporeal circulation

Y. Denizot,[1] P. Fixe,[1] E. Cornu[2] and N. Nathan[1,CA]

Departments of [1]Anaesthesia and [2]Cardiovascular Surgery, CHRU Dupuytren, 2 Avenue Martin Luther King, 87042 Limoges, France

[CA]Corresponding Author
Fax: (+33) 05 55 05 67 92

**Leukocytosis and thrombocytopenia occur during cardiopulmonary bypass (CPB) with extracorporeal circulation (ECC). Elevated circulating concentrations of macrophage colony-stimulating factor (M-CSF) are reported during thrombocytopenia and leukopenia of different origins. We have assessed M-CSF concentrations in 40 patients undergoing CPB with ECC. Plasma M-CSF concentrations were stable during ECC and increased at the 6th (7.3 ± 0.7 IU/μg protein) and 24th (8.6 ± 0.8 IU/μg protein) postoperative hour compared with pre-ECC values (4.9 ± 0.5 IU/μg protein). A deep thrombocytopenia was found during ECC and until the 24th postoperative hour. A drop of leukocyte counts was found during ECC followed by an increase after ECC weaning. While no correlation was found between M-CSF concentrations and the leukocyte counts, M-CSF values were positively correlated with platelet counts only before and during ECC. Thus, M-CSF is not implicated in the thrombocytopenia and the leukopenia generated during CPB with ECC. However the elevated levels of M-CSFa few hours after the end of ECC might play a role in the inflammatory process often observed after CPB.**

**Key words:** Cardiopulmonary bypass, CEC, Inflammation, M-CSF

### Introduction

Cardiopulmonary bypass (CPB) with extracorporeal circulation (ECC) results in major haematological changes including leukocytosis and thrombocytopenia.[1] The release of several inflammatory compounds including cytokines, eicosanoids, platelet-activating factor (PAF), and activation of the coagulation and the complement cascade are suspected to play a role in these haematological disorders and in the pathogenesis of the inflammatory syndrome observed after CPB.[2–6]

Monocyte/macrophage activation accompanies CPB.[7] Macrophage colony-stimulating factor (M-CSF), also known as CSF-1, is a major regulator of the production as well as functional state of cells of the mononuclear-phagocytic lineage.[8,9] M-CSF is produced by numerous cell types such as endothelial cells, monocytes/macrophages, T- and B-cells spontaneously or after induction.[8–10] M-CSF acts on mature monocytes/macrophages, stimulating production of tumour necrosis factor (TNF), interferon, interleukin-6 (IL6), prostaglandins and CSFs.[11–13] Some reports have highlighted the role of M-CSF in the pathogenesis of thrombocytopenia and leukopenia of various origins.[14–16] The purpose of this study was to assess plasma M-CSF concentrations during and after cardiac surgery with ECC. We also investigated the relationship between platelet and leukocyte counts and M-CSF levels during and after CPB.

### Patients and Methods

After ethical committee approval, 40 patients scheduled to undergo coronary artery bypass graft were included in this prospective study. All the patients gave written informed consent.

### Anaesthesia

All patients had a preoperative ejection fraction above 40%. Upon arrival on operating room, all patients received their usual oral cardiac medication, flunitrazepam (2 mg) and morphine (10 mg) intramuscularly. Anaesthesia was induced and maintained with titrated doses of fentanyl and flunitrazepam. Muscular relaxation was achieved with pancuronium (0.1 mg/kg). Bubble or membrane oxygenators were used according to the planed number of graft and surgical difficulties. The blood was harvested

© 1996 Rapid Science Publishers from surgical field and from the cell saver at the end of ECC and reinfused to all patients. All patients (except two with uneventful surgery) received high doses aprotinin. All the patients were managed by the same surgical anaesthetic team.

### Blood sampling and haematologic measurements

Blood samples were collected from the radial artery catheter at the following times: before vascular cannulation and after opening the chest ($T_0$), during ECC just before ($T_1$) and after cross-clamp release ($T_2$), after weaning from ECC ($T_3$), at the 6th ($T_4$) and 24th post-operative hour ($T_5$) (Fig. 1). Two ml of blood were placed in EDTA then centrifuged to obtain plasma which was stored at −80°C until assay. Plasma samples were also collected from 36 healthy individuals to assess plasma M-CSF concentrations in controls. Platelet and leukocyte counts were determined in the radial artery blood at $T_0$, $T_1$, $T_3$, $T_4$ and $T_5$.

Haematological complications were biologically determined at $T_3$, $T_4$ and $T_5$ as follows: APTT values above 1.5, fibrinogen levels $< 1$ g/l, factor V levels $< 30\%$, and platelet count $< 70\,000$ elements/mm$^3$. Clinical bleeding was considered when blood loss reached values above 100 ml/h. Haematological complications were graded from 0 (absence) to 3 [biological trouble and significant bleeding leading (grade 3) or not (grade 2) to reoperation]. Grade 1 haematological complications were assumed when biological abnormalities were present without clinical bleeding.

### M-CSF assay

M-CSF production was determined by a self-made sandwich EIA procedure previously described.[17,18] Briefly, polyclonal anti-human M-CSF immunoglobulins were coated on 96-well microtitre plates. Standard dilutions of M-CSF (400 to 6 international units per ml (IU/ml) of human M-CSF, international standard, NIBSC, Potters Bar, UK) and samples were transferred into wells and incubated overnight at 4°C. Plates were washed and anti-M-CSF-biotin conjugate was added to all wells and incubated overnight at 4°C. Plates were washed and avidine phosphatase (1 μg/ml) was added to wells and incubated for 1 h at 37°C. The revelation was performed with para-nitrophenyl-phosphate disodium (4 g/ml) for 15 min at 37°C. The reaction was blocked by addition of 50 μl of NaOH 10N to the wells. Colour development was measured at OD 405 nm. M-CSF levels in samples were calculated with respect to the calibration curve obtained with standard M-CSF. The sensitivity of the assay enables detection of plasma M-CSF levels as low as 10 IU/ml. Plasma M-CSF concentrations were expressed either as IU/ml for controls and for patients before surgery or as IU per microgram of protein content (IU/μg) to exclude the influence of haemodilution for operated patients. The proteinaemia was determined at each time of blood sampling by the BCA Protein Assay Reagent (Pierce, Rockford, IL).

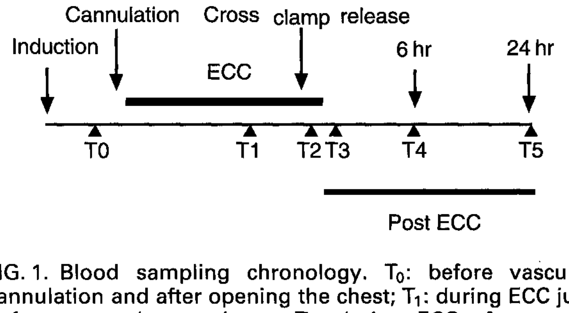

FIG. 1. Blood sampling chronology. $T_0$: before vascular cannulation and after opening the chest; $T_1$: during ECC just before cross-clamp release; $T_2$: during ECC after cross-clamp release; $T_3$: after weaning from ECC; $T_4$: the 6th postoperative hour; $T_5$: the 24th postoperative hour.

### Statistical analysis

Results are expressed as mean ± SEM. Statistical analysis was performed by Kruskal Wallis analysis and Mann–Whitney *U*-test. A $p < 0.05$ was considered significant. Statistical analysis was performed using the statistics package 'statgraphic'. Correlations between M-CSF levels and platelet or leukocyte counts were calculated by linear regression analysis.

### Results

The demographic data of patients are shown in Table 1. Plasma M-CSF concentrations of coronary patients before ECC (223.7 ± 20.6 IU/ml, $n = 40$) were not statistically ($p > 0.05$, Mann–Whitney *U*-test) different compared with healthy individuals (198.3 ± 17.4 IU/ml, $n = 36$).

A significant ($p < 0.01$) drop in platelet and leukocyte counts was observed in the radial blood during ECC (Fig. 2). Platelet counts remained low at the 6th and 24th postoperative hour. By contrast leukocyte counts increased significantly ($p < 0.01$) after weaning of ECC and at the 6th and 24th postoperative hour. As shown in Fig. 2, plasma M-CSF concentrations were stable during ECC. Elevated levels ($p <$ 0.01, Mann–Whitney *U*-test) of plasma M-CSF

**Table 1.** Demographic data (mean ± SEM)

| | Patients ($n = 40$) |
|---|---|
| Age (years | 67 ± 1 |
| Weight (kg) | 74 ± 2 |
| Body surface area ($m^2$) | 1.84 ± 0.03 |
| Ejection fraction (%) | 58.6 ± 2.7 ($n = 33$) |
| Preoperative left ventricle diastolic pressure (mmHg) | 16.1 ± 1.6 ($n = 36$) |
| Number of grafts | 3.0 ± 0.1 |
| ECC duration | 104 ± 6 |
| Heparin doses (mg) | 357 ± 11 |
| Protamine doses (mg) | 267 ± 9 |
| Peroperative blood loss (ml) | 603 ± 47 |

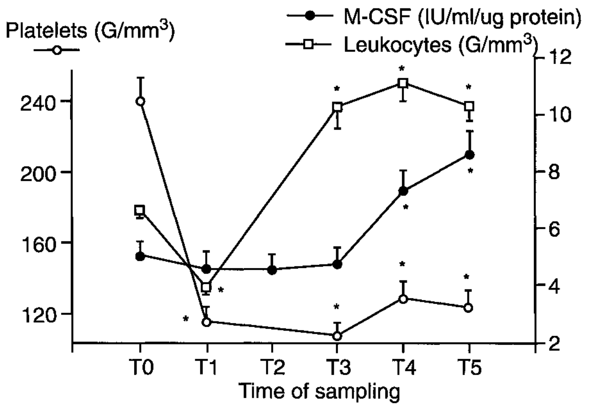

FIG. 2. Plasma M-CSF concentrations (●), platelet (○) and leukocyte (□) counts during and after CPB. Platelet and leukocyte counts are expressed in g/ml. M-CSF concentrations are expressed in IU/ml/µg protein. Mean ± SEM. * $p < 0.01$ compared with $T_0$ (Mann–Whitney $U$-test).

were found at the 6th (7.3 ± 0.7 IU/ml/µg protein) and 24th (8.6 ± 0.8 IU/ml/µg protein) postoperative hour compared with $T_0$ (4.9 ± 0.5 IU/ml/µg protein).

A positive correlation was observed before ($T_0$) and during ECC ($T_1$) between platelet counts and M-CSF concentrations (Table 2). After cross-clamp release no correlation was found between platelet counts and M-CSF concentrations. No correlation was found between M-CSF concentrations and leukocyte counts at any blood sampling time (Table 2).

No difference was documented between M-CSF concentrations according to the grade of haematological complications at $T_3$, $T_4$ and $T_5$ (Fig. 3).

### Discussion

This study shows that plasma M-CSF concentrations are stable during ECC and increase at the 6th and 24th postoperative hour compared with pre-ECC values. While lipidic mediators are produced chiefly during ECC,[7,8] elevated cytokine concentrations are often found after few hours.[5,6,10] The time-course of M-CSF concentrations during cardiac surgery fits well with those of the other cytokines so far assessed. We may speculate that the monocytes, lymphocytes, and polymorphonuclear neutrophils 'primed' by CPB might be implicated in these elevated M-CSF concentrations. However a lowering M-CSF degradation might also explain our results. M-CSF receptors is almost totally cleared from the

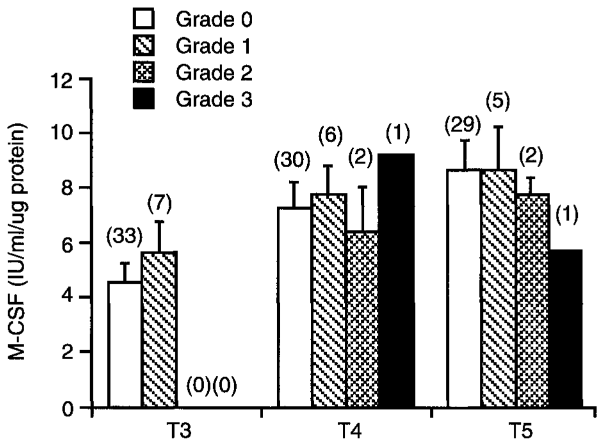

FIG. 3. Plasma M-CSF after ECC ($T_3$), at the 6th ($T_4$) and 24th ($T_5$) postoperative hour in relation to the grade of haematological complication. Grade 0: no trouble; Grade 1: haematological complication without clinical bleeding; Grade 2: haematological complication with bleeding; Grade 3: haematological complication with bleeding leading to reoperation. M-CSF concentrations are expressed in IU/ml/µg protein. ($n$): number of patients.

**Table 2.** Correlations between plasma M-CSF levels and platelet and leukocyte counts during and after CPB. (n): number of patients

| | Time of blood sampling | | | | |
|---|---|---|---|---|---|
| | $T_0$ ($n = 32$) | $T_1$ ($n = 32$) | $T_3$ ($n = 31$) | $T_4$ ($n = 33$) | $T_5$ ($n = 33$) |
| Platelets | $r = 0.39$ | $r = 0.39$ | $r = 0.29$ | $r = 0.02$ | $r = -0.04$ |
| | $p = 0.020$ | $p = 0.022$ | $p = 0.099$ | $p = 0.88$ | $p = 0.81$ |
| Leukocytes | $r = 0.21$ | $r = 0.24$ | $r = -0.15$ | $r = -0.12$ | $r = 0.04$ |
| | $p = 0.22$ | $p = 0.17$ | $p = 0.41$ | $p = 0.46$ | $p = 0.78$ |

circulation by specific M-CSF receptors on hepatic and splenic macrophages.[11,12] A down-regulation of M-CSF receptors at the surface of macrophages has been reported in response to inflammatory mediators and cytokines.[19] Such a phenomenon might also explain the elevated M-CSF levels after CPB. The production of M-CSF by peripheral blood leukocytes of patients undergoing CPB with ECC deserves now to be assessed.

M-CSF is suspected to play a role in the fall of platelet counts during pregnancy,[14] and in patients with immune thrombocytopenic purpura.[15] In this study M-CSF levels are stable during ECC. While M-CSF levels are positively correlated with platelet counts at the beginning of ECC, no correlation is found with leukocyte counts. Thus, M-CSF is not implicated in the thrombocytopenia and the leukopenia which occur at the beginning of ECC. Various mediators released during coronary reflow amplify vascular cell margination and may participate to vascular obstruction and to the no-reflow phenomenon.[7] Immediately after ECC, plasma M-CSF concentrations are similar to pre-ECC values denying the involvement of M-CSF in the haematologic changes generated during the coronary reflow. After ECC the increase of leukocyte counts occurs before the increase of M-CSF concentration denying its role in the post-ECC leukocytosis. In addition no difference could be documented between M-CSF concentrations after ECC according to the grade of haematological complications.

The concept that there is a marked synergy between the effects of the various mediators released during cardiac surgery is now widely accepted.[2] A combination of small amounts of them may be more active than a large release of one by itself. M-CSF might play a role in the inflammatory process observed after cardiac surgery since the key cells of the inflammatory response to CPB (i.e. monocytes, polymorphonuclear neutrophils and lymphocytes) produce M-CSF in response to cytokines and produce cytokines in response to M-CSF.[8–13] In addition elevated circulating amounts of granulocyte-CSF (G-CSF) have been recently found after cardiac surgery,[20] strengthening that the involvement of growth factors after CPB deserves further evaluations.

### References

1. Pearl RG, Sladen RN, Rosenthal MH. Hematologic effects of cardiac and noncardiac surgery. *J Cardiothorac Anesth* 1987; **1**: 205–209.
2. Casey LC. Role of cytokines in the pathogenesis of cardiopulmonary-induced multisystem organ failure. *Ann Thorac Surg* 1993; **56**: S92–S96.
3. Steinberg BM, Grossi EA, Schwartz DS, *et al.* Heparin bonding of bypass circuits reduces cytokine release during cardiopulmonary bypass. *Ann Thorac Surg* 1995; **60**: 525–529.
4. Ylikorkala O, Saarela E, Viinikka L. Increased prostacyclin and thromboxane production in man during cardiopulmanary bypass. *J Thorac Cardiovasc Surg* 1981; **82**: 245–247.
5. Faymonville ME, Deby-Dupont G, Larbuisson R, *et al.* Prostaglandin E2, prostacyclin, and thromboxane changes during nonpulsatile cardiopulmonary bypass in humans. *J Thorac Cardiovasc Surg* 1986; **91**: 858–866.
6. Nathan N, Denizot Y, Feiss P, Laskar M, Arnoux B, Benveniste J. Variations of blood PAF-acether levels during coronary artery surgery. *J Cardiothor Vas Anesth* 1992; **6**: 692–696.
7. Butler J, Rocker GM, Westaby S. Inflammatory response to cardiopulmonary bypass. *Ann Thorac Surg* 1993; **55**: 552–559.
8. Stanley ER, Berg KL, Einstein DB, Lee PSW, Yeung YG. The biology and action of colony stimulating factor-1. *Stem Cells* 1994; **12** (suppl 1): 15–25.
9. Praloran V. Structure, biosynthesis and biological roles of monocyte-macrophage colony stimulating factor (CSF-1 or M-CSF). *Nouv Rev Fr Hematol* 1991; **33**: 323–333.
10. Lindemann A, Riedel D, Oster W, Ziegler-Heitbrock HWL, Mertelsmann R, Herrmann F. Granulocyte-macrophage colony-stimulating factor induces cytokine secretion by human polymorphonuclear leukocytes. *J Clin Invest* 1989; **83**: 1308–1312.
11. Warren MK, Ralph P. Macrophage growth factor CSF-1 stimulates human monocyte production of interferon, tumor necrosis factor, and colony stimulating activity. *J Immunol* 1986; **137**: 2281–2285.
12. Kurland JI, Pelus LM, Ralph P, Bockman RS, Moore MAS. Induction of prostaglandin E synthesis in normal and neoplastic macrophages: role for colony-stimulating factor(s) distinct from effects on myeloid progenitor cell proliferation. *Proc Natl Acad Sci USA* 1979; **76**: 2326–2330.
13. Temeles DS, McGrath HE, Kittler ELW, *et al.* Cytokine expression from bone marrow derived macrophages. *Exp Hematol* 1993; **21**: 388–393.
14. Praloran V, Coupey L, Donnard M, Berrada L, Naud MF. Elevation of serum M-CSF concentrations during pregnancy and ovarian hyperstimulation. *Br J Haematol* 1994; **86**: 675–677.
15. Yong K, Salooja N, Donahue RE, Hegde U, Linch DC. Human macrophage colony-stimulating factor levels are elevated in pregnancy and in immune thrombocytopenia. *Blood* 1992; **80**: 2897–2902.
16. Jewell AP, Yong KL, Worman CP, Tsakona CP, Giles FJ, Goldstone AH. Serum macrophage colony-stimulating factor (M-CSF) levels correlate with clinical response to interferon-alpha in patients with early-stage B-CLL. *Br J Haematol* 1994; **86**: 441–443.
17. Dupuis F, Denizot Y, Fixe P, Dulery C, Praloran V. PAF and haematopoiesis. X. Macrophage colony-stimulating factor and granulocyte macrophage colony-stimulating factor enhance platelet-activating factor acetylhydrolase production by human blood-derived macrophages. *Biochim Biophys Acta* 1996; **1311**: 27–32.
18. Coupey L, Berrada L, Gascan H, Godard A, Praloran V. High titre anticytokine antibodies obtained by intralymphnode immunization with low amounts of antigen. *Cytokine* 1993; **5**: 564–569.
19. Dello Sbarba P, Cipolleschi MG, Buscher D, Baccarini M. Modulation of the macrophage colony stimulating factor receptor and control of growth in activated macrophages. *Minerva Biotec* 1994; **6**: 146–158.
20. Iwasaka H, Unoshima M, Noguchi T, Kitano T, Taniguchi K, Honda N. Neutrophilia and granulocyte colony-stimulating factor levels after cardiopulmonary bypass. *Anesthesiology* 1995; **83**: A95 (abstract).

ACKNOWLEDGEMENTS. P.F. is the recipient of a grant from the 'Ligue Nationale Contre le Cancer' (Comité de la Haute Vienne).

**Received 1 August 1996; accepted 28 August 1996**
